# Active learning-based systematic reviewing using switching classification models: the case of the onset, maintenance, and relapse of depressive disorders

**DOI:** 10.3389/frma.2023.1178181

**Published:** 2023-05-16

**Authors:** Jelle Jasper Teijema, Laura Hofstee, Marlies Brouwer, Jonathan de Bruin, Gerbrich Ferdinands, Jan de Boer, Pablo Vizan, Sofie van den Brand, Claudi Bockting, Rens van de Schoot, Ayoub Bagheri

**Affiliations:** ^1^Department of Methodology and Statistics, Faculty of Social and Behavioral Sciences, Utrecht University, Utrecht, Netherlands; ^2^Amsterdam UMC, Department of Psychiatry and Centre for Urban Mental Health, University of Amsterdam, Amsterdam, Netherlands; ^3^Department of Research and Data Management Services, Information Technology Services, Utrecht University, Utrecht, Netherlands; ^4^Utrecht University Library, Utrecht University, Utrecht, Netherlands

**Keywords:** active learning, systematic review, convolutional neural network, model switching, simulations, work saved over sampling

## Abstract

**Introduction:**

This study examines the performance of active learning-aided systematic reviews using a deep learning-based model compared to traditional machine learning approaches, and explores the potential benefits of model-switching strategies.

**Methods:**

Comprising four parts, the study: 1) analyzes the performance and stability of active learning-aided systematic review; 2) implements a convolutional neural network classifier; 3) compares classifier and feature extractor performance; and 4) investigates the impact of model-switching strategies on review performance.

**Results:**

Lighter models perform well in early simulation stages, while other models show increased performance in later stages. Model-switching strategies generally improve performance compared to using the default classification model alone.

**Discussion:**

The study's findings support the use of model-switching strategies in active learning-based systematic review workflows. It is advised to begin the review with a light model, such as Naïve Bayes or logistic regression, and switch to a heavier classification model based on a heuristic rule when needed.

## 1. Introduction

Researchers write systematic reviews and conduct meta-analyses to provide an exhaustive summary of a specific scientific field, providing essential, comprehensive overviews of relevant topics (Moher et al., 2015). Each systematic review requires manually screening hundreds to tens of thousands of records, only to include a few relevant papers. Few relevant records result in a highly imbalanced dataset, with relevant records being very sparse (i.e., usually < 5%). The classification process can be significantly improved by utilizing an active learning-based systematic review pipeline (Settles, 2012). This pipeline uses machine learning models to help users screen the records that are most likely to be relevant (also called certainty-based sampling) and, simultaneously, enhance the model to be more adept at finding and presenting those relevant records to the user. Active learning has been shown to outperform random reading using various feature extraction techniques and classifiers (van de Brand and van de Schoot, 2021). These techniques apply many different forms of processing, each having its advantages and disadvantages (Naseem et al., 2021).

Human-computer interaction plays a crucial role in the systematic review process, particularly in computer-aided systems where collaboration between human reviewers and machine learning models is essential for improved efficiency and accuracy. In linguistics, computer-aided systems improve functional style identification and correction in texts (Savchenko and Lazebnik, 2022); in science education, they help measure complex science reasoning (Liu et al., 2008); in healthcare, they guide the research-to-policy and practice cycle (Best et al., 2009); in software development, they boost effectiveness and reduce defects (Tiwana, 2004); and in physics-informed symbolic regression, they advance the discovery of meaningful symbolic expressions (Keren et al., 2023). These works highlight the importance of designing user-friendly interfaces, providing accurate and timely feedback, and understanding the cognitive processes involved in human decision-making when interacting with machine learning models. By building on these insights, our current work aims to research the active learning-based pipeline that effectively combines the strengths of both human expertise and machine learning algorithms, ultimately enhancing the overall systematic review process.

The active learning-based pipeline used for aiding systematic reviews consists of different steps turning natural language into practical representations that can be used to make predictions on relevance. Computational time is of great importance in the case of applying active learning to the use-case of systematic reviewing. That is, while a human is screening the next record in the queue, based on the model's relevance estimates of the previous iteration, a model is trained in the back-end. Ideally, the model should be done with re-training before the human annotator (reviewer) has finished reading the current record so that the next abstract shown to them is the result of the new model. Therefore, limited computational time is vital in the case of systematic reviewing.

There is a wide range of algorithms for text classification, from logistic regression to naive Bayes, a probabilistic classifier, and more advanced machine learning techniques like support vector machine (SVM) or decision tree. However, the interconnectivity of records is not an exact science. Similar records might be found by comparing the record vocabulary, but not in all cases. Records can be hard to find due to concept ambiguity, the different angles from which a subject can be studied, and changes in the meaning of a concept over time, known as concept drift (Chen et al., 2012; Gama et al., 2014). These characteristics make it difficult for standard techniques to learn which texts are relevant, and the algorithms must “dig deeper” into a text to find its essence (Goodfellow et al., 2016). Deep learning networks, such as convolutional or recurrent neural networks, are better at finding complex connections within data when compared to classical machine learning algorithms. According to previous research (Rolnick and Tegmark, 2017), it is exponentially easier to approximate sparse multivariate polynomials with deep neural networks compared to shallow networks performing the same task. The term deep learning references the multiple layers a deep neural network has. Where shallow networks only have one or two layers, a deep learning network can have many layers, only restricted by the computing power available. These deep layers are where the complex connections are found.

A convolutional neural network (CNN)-based approach is proposed to implement a deep neural network. This type of neural network is often successfully used in text classification tasks (Collobert and Weston, 2008; Hughes et al., 2017) but is, as far as is known, never used in aid of systematic reviews. The convolutional layers found in CNNs are a specialized and efficient neural network foundation, more so than standard dense layers are. In dense layers (often called a “fully connected layer”), each neuron is connected to every neuron in the layer before, making them expensive to compute. Convolution layers are only connected to a few neighboring neurons, and the weights are the same for each connection. Having fewer connections makes convolutional layers cheaper to compute than dense layers. These local connections extract information from input data where features are locally related. This makes convolutional layers strong in text-related neural networks and thus applicable for the systematic review process.

However, CNN models require much more training data (Montavon et al., 2012) and, as Alwosheel et al. show, the performance of neural networks in classification problems increases with dataset sample size (Alwosheel et al., 2018). For example, Giga5—a commonly used dataset for training deep learning models—contains almost 10 million documents (Parker et al., 2011). A study shows that shallow neural networks can achieve better error rates than deep neural networks for text classification in some situations, with deep neural networks outperforming shallower models when the dataset was 2.6 million documents but performing poorly when training data was 120 K documents (Johnson et al., 2016). Systematic reviews usually have a few thousand records (De Boer et al., 2021). Moreover, active learning for systematic reviewing can already start with only a few labeled records as training data for the first iteration of the model (van de Schoot et al., 2021a). Therefore, starting with a CNN model in the first couple of iterations is not expected to result in a good performance. Only when enough labels are available, a CNN might outperform shallow classifiers. Therefore, we propose to start with a shallow classifier and only switch to a CNN model when enough labeling decisions are available for training in the model.

Another reason why switching to a CNN model might be beneficial is that often the first set of relevant records can easily be found, whereas the last records take significant effort for the active learning model (van de Schoot et al., 2021a). The last-to-find records might therefore be semantically different compared to the records found in the early phase. The distribution of relevant records can form clusters if the dataset spans multiple semantic clusters. If the classifier has found many records from one cluster, it can be over-fit to find records from other clusters. The classifier can only begin identifying additional records within a cluster once a record from that cluster has been located. These clusters can create some difficult situations during classifying.

Therefore, for the current study, we first demonstrate the advantage of using Active Learning over manual screening for a large labeled dataset of >46 K records. We computed the Work Saved over Sampling (WSS) to evaluate the performance compared to random reading. We also computed the average time to discovery (ATD) of the relevant records to show there are last-to-find papers. Then, we present the results of the original meta-analysis (Brouwer et al., 2019) and test what would have happened if the last-to-find papers were not taken into account. In a second study, we developed an optimized convolutional neural network. In the third study, we compared the performance in terms of WSS and computational time for different combinations of classifiers (NB, SVM, LR, RF, and two-layer-NN) with feature extraction techniques (TF-IDF, Doc2Vec, and SBert), and compared these to the newly developed 17-layer CNN model. In the fourth study, we examined if switching from a classical algorithm to a neural network increases performance compared to the best performing method of simulation study 3. All simulations were carried out with the simulation mode of the open-source software ASReview (van de Schoot et al., 2021b). For reproducibility, all scripts and output are available on Github (Teijema et al., 2022).

### 1.1. Data

The dataset used in this study comes from a systematic review-based meta-analysis focusing on the evidence for leading psychological and biological theories on the onset, maintenance, and relapse of depressive disorders (Brouwer et al., 2019; Kennis et al., 2020; Fu et al., 2021). For this project, 18 researchers screened approximately 150,000 records for relevance, which took them 3 years. Within a sub-project of this project, the researchers screened over 46 thousand records for a question on psychological theories of depressive relapse. They identified only 63 eligible papers for the final meta-analysis (0.13% inclusion rate). In this project, only longitudinal and prospective studies were included to establish a hypothesized causality between the theories and depressive disorders for five leading psychological theories of relapse and recurrence of major depressive disorder: cognitive, diathesis-stress, behavioral, psychodynamic, and personality-based.

In the study by Brouwer et al. (2019), information about the dataset construction can be found, providing insights into the methodology and approach used by the researchers. For an understanding of the search strategy and selection process of relevant records, readers can refer to Appendix B of Brouwer et al. (2019), which contains the search keys used during the literature review. This information is important for replicating the process or conducting further research on the topic.

To establish a direct link and robust effects, any factor derived from one of the five theories needed to be assessed before the relapse or recurrence of major depressive disorder. The status of the disorder was required to be at least at two-time points prospectively through a clinical interview or expert opinion. The goal was to investigate the leading psychological theories, and thus all factors derived from that leading theory were pooled and analyzed. The primary outcome was the effect of the theory-derived factor on the risk of relapse or recurrence of major depressive disorder. The effect sizes Hazard Ratios (HR) and Odds Ratios (OR) for all factors were calculated using reported statistics from each study with the software program Comprehensive Meta-Analysis (Borenstein et al., 2021). The effect sizes were pooled using random-effects models, and the results were published (Brouwer et al., 2019). All pooled odds ratios and hazard ratios are available on the Open Science Framework (Brouwer and van de Schoot, 2021).

### 1.2. Pre- and postprocessing

The data (Brouwer et al., 2019) consist of the title and abstracts of all the records identified in the search and their respective labeling decision (i.e., relevant/irrelevant). Before this data could be used for simulations, it needed to be pre-processed. The researchers used several spreadsheets to manage the enormous number of records and labeling decisions. However, the simulations required a single file with three columns (title, abstract, labeling decision) and a low percentage of missing data. Therefore all original files were merged, and missing abstracts were added. A description of the entire preprocessing procedure is found on the Open Science Framework (Brouwer et al., 2021).

For this study, the pre-processing procedure was continued on the dataset with the addition of stricter deduplication rules, to increase the cleanliness of the dataset. On top of that, missing DOIs were obtained, and noisy labels were corrected in two rounds of quality checks. The deduplication scripts are available on Zenodo (van den Brand et al., 2021).

The exact number of records in the post-processed dataset is 46,376, of which 63 were included in the final meta-analysis. This ratio results in a relevance rate of less than 0.14%. The average abstract contains 218 words.

## 2. Study 1—Active learning-aided systematic reviewing

The purpose of the first study is to increase the confidence in active learning for systematic reviews. It investigates the work saved by using active learning, expressed in the WSS metric (Work Saved over Sampling). This metric is calculated from the ratio of effort saved compared to screening records randomly. The study also investigates the stability of the active learning aided systematic review by measuring the impact of skipping the last-to-find records of the original meta-analyses calculations.

When using the active learning pipeline, not all records are screened. This method saves time but introduces a chance that relevant records are not suggested for screening, although it is unknown if this impact is equal to or smaller than the impact of screening fatigue losses. If the effect of missing the last-to-find records is low, this will lower the perceived risk of using this method. Study 1 aims to address this risk by answering the following research questions:

**RQ1.1** How much time would the active learning application have saved during the systematic review that resulted in the Brouwer et al. dataset (Brouwer et al., 2019)?

**RQ1.2** What effect does the selected prior knowledge have on the average time to discover the relevant records?

**RQ1.3** What is the impact of failing to discover the last-to-find records in the systematic review from the Brouwer et al. dataset (Brouwer et al., 2019)?

### 2.1. Method

Using a pre-labeled dataset, such as the one used in this study, the labeling via the active-learning pipeline can be simulated, replicating the choices made by the reviewer, and training the model as it would during authentic use. Using these simulations, different models can be compared on how many records would have been found before the user stops reviewing. To answer RQ1.1 and RQ1.2, a simulation was run for each relevant record, and differences between simulation records were examined.

For RQ1.3, the median last-to-find records were removed from the meta-analysis, and the Hazard Ratios (HR) and Odds Ratios (OR) were re-calculated.

### 2.2. Setup

In study 1, we utilized the dataset from Brouwer et al. to assess the efficiency of our active learning-based approach.

The simulation study was conducted with the default settings of ASReview v0.18 (van de Schoot et al., 2021c). The default settings are classification by naïve Bayes combined with term frequency-inverse document frequency (TF-IDF) feature extraction approach for the active learning model. The number of runs was set equal to the number of inclusions in the dataset (i.e., 63). Every run started with training data consisting of only one relevant and ten randomly chosen irrelevant records (held constant across runs).

Randomly screening records and screening records using the active learning pipeline are compared using the WSS metric. This metric is defined as the percentage of papers a researcher does not have to screen. WSS@95% is measured at a recall level of 95%, meaning that it reflects the amount of work saved by using active learning at the cost of failing to identify 5% of relevant publications. Note that humans typically fail to find about 10% due to screening fatigue (Wang et al., 2020).

For the 63 included records, the Average Time to Discovery (ATD) was computed by taking the average of the time to discovery of all relevant records (Ferdinands et al., 2020). The time to discovery for a given relevant publication was computed as the number of records needed to screen to detect this record. All code to reproduce the simulation results and the output of the simulations can be found at (Ferdinands et al., 2021).

Finally, the original meta-analysis was redone, excluding the 5 and 10% last-to-find records (i.e., with the highest ATD). The results of the original meta-analysis and the new results are available on the Open Science Framework (Brouwer and van de Schoot, 2021).

### 2.3. Results

Our findings build upon the Brouwer et al. (2019) dataset by demonstrating that active learning can significantly reduce screening time and efficiently identify relevant records in a systematic review. This suggests that our approach could potentially enhance the methodology used in Brouwer et al. (2019) study by increasing the speed and accuracy of the review process.

**Figure 1:**
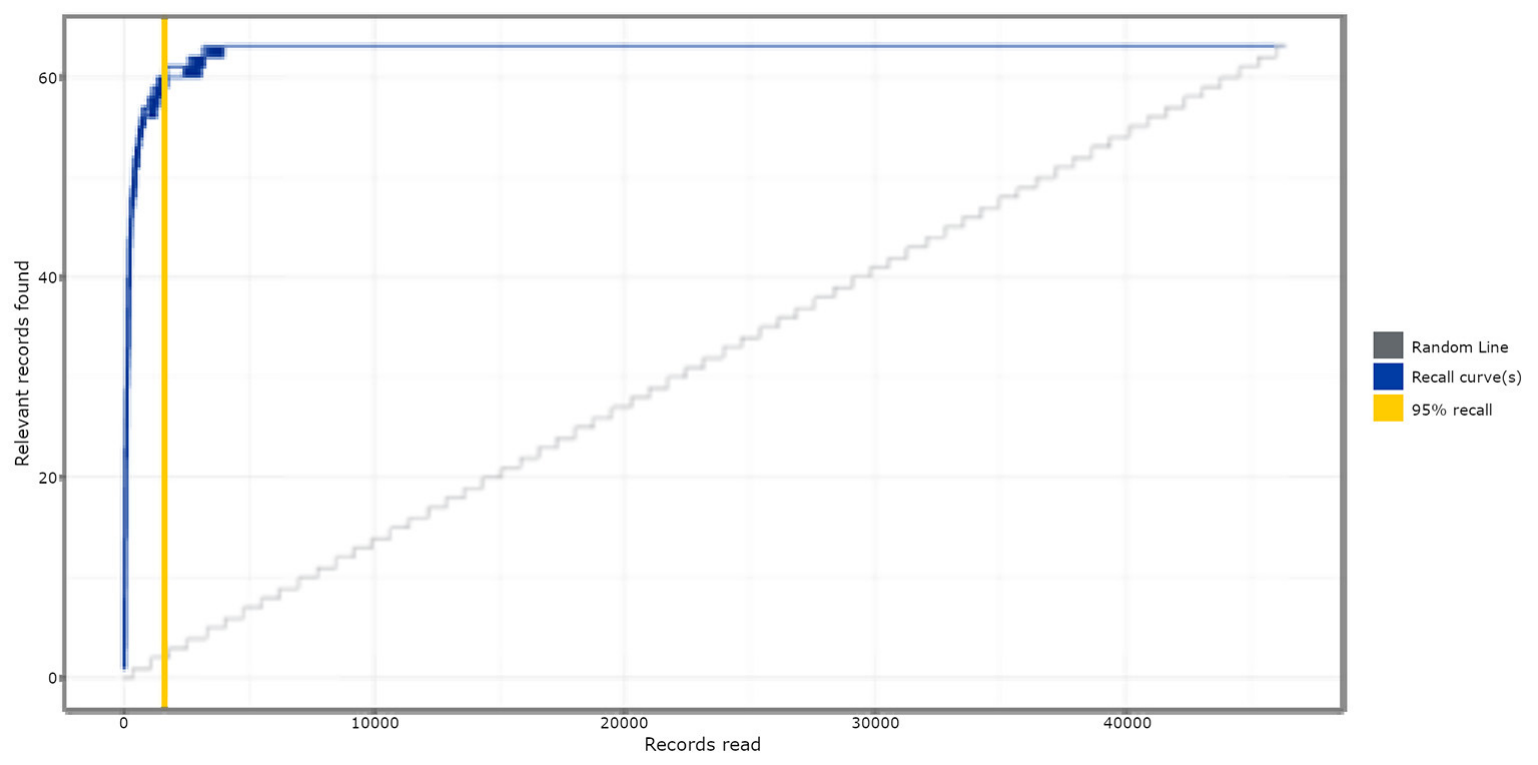
Simulation results of study 1. The absolute amount of relevant publications found is displayed on the y-axis, and the absolute amount of screened publications is on the x-axis. The solid blue lines are a combination of all the Recall curves, representing the relevant records found as a function of the screened publications for each of the 63 ran simulations.

Figure 1 shows the simulation results of study 1, comparing the active learning-based approach to random reviewing, when testing on the Brouwer et al. (2019) dataset. It appeared that with active learning, on average, 92% (SD = 0.18; Min/max = 91.65/92.25) of the screening time (WSS) could have been saved compared to reading records at random. After screening only 5% of the total number of records, already 95% (SD = 0.35; Min/max = 95.16/96.77) of the relevant records were found. Based on these results, active learning shows significant time-saving potential compared to random reading.

Results show that excluding the 5 or 10% of last-to-find records from the analysis has no impact on analysis results. The conclusions drawn from these papers would have been similar when excluding the last. Even when excluding the last 10% of found records, the results overall remained alike for the analyses on time to relapse (Hazard Ratio) with an insignificant difference in pooled effects. For the odds ratios, the primary analyses (pooled effect sizes for the five leading theories) remained similar and differed only numerically for some subgroup analyses. When analyzing the effect of depressive symptoms on the predictive value of behavioral theories on the odds of depressive relapse, the effects changed from “just”-significant to “just” not-significant (Odds Ratio), which was due to one missing study. All other results were similar to the initial results. According to the original authors, neither the original paper's conclusion nor the clinical advice would have changed had the last-to-found records not been included in the review, indicating that these records are not of special relevance to the dataset.

Our results address the research questions as follows: For RQ1.1, we found that active learning saved an average of 92% of screening time compared to reading records at random during the systematic review that resulted in the Brouwer et al. dataset. In terms of RQ1.2, we observed that the prior knowledge had no impact on the average time to discover relevant records in the systematic review. Lastly, addressing RQ1.3, our analysis revealed that failing to discover the last-to-find records in the systematic review from the Brouwer et al. dataset did not impact the analysis results or clinical advice, indicating these records were not of special relevance to the dataset. This suggests that stopping the review process earlier does not carry any particular risk associated with missing critical information, as the last records were not found to be more significant or influential compared to the others.

## 3. Study 2—Development of deep neural networks

In ASReview, the implemented neural network is a feed-forward two-layer-based model (van de Schoot et al., 2021a). The goal of the second study is to propose an optimized deep neural network as a classification model. For this study, the chosen implementation of deep learning was a convolutional neural network consisting of 17 hidden layers. CNNs have been proven to be very effective in text classification problems (Hughes et al., 2017). No such neural network has been used for active learning in systematic reviewing before, to the best of our knowledge. However, this type of neural network is often used in hierarchical classification problems such as ordering records on relevance (Jaderberg et al., 2016). The convolutional layers found in a CNN have fewer connections than the fully connected layers often found in neural networks. The fewer connections and weights make convolutional layers cheaper in terms of memory and compute power needed. Their structure is designed not to be fully connected, opting to find local patterns first and combine them later. Reduced computational power is an essential feature, as every iteration in the classification re-trains the neural network. On the other hand, a fully connected neural network with a similar amount of layers as the implemented network would not be a feasible solution when considering the computational time in relation to the active learning pipeline.

In the second study, the only objective was to develop the CNN network. This leads to the following research question:

**RQ2.1:** Can a convolutional neural network be effective in text classification for the purpose of active learning in systematic review?

### 3.1. Setup

**Figure 2:**
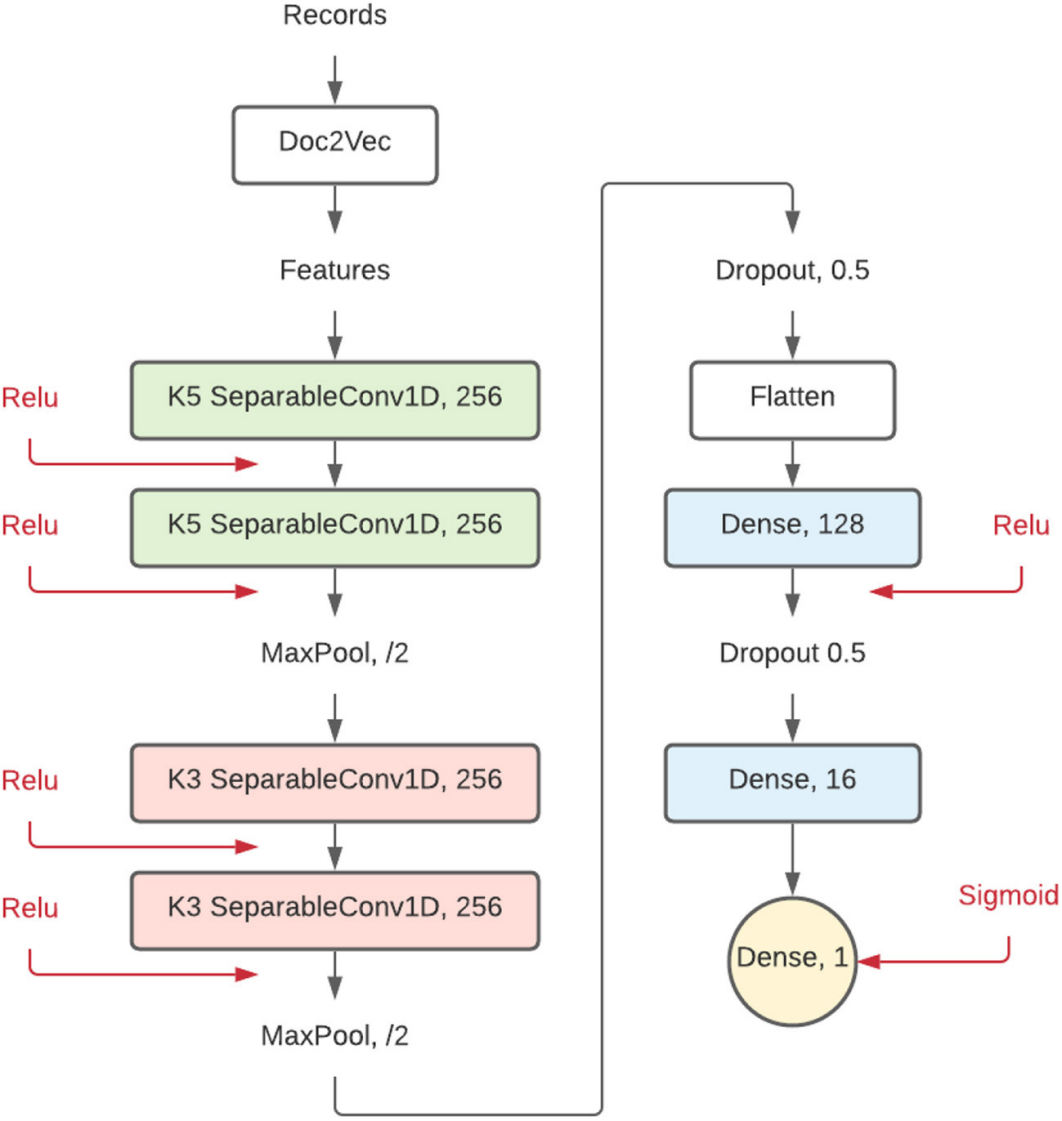
Proposed convolutional neural network model. Each element represents a different layer of the neural network. Numbers behind the layer title are settings for that layer.

The model implemented in this study has a comparable structure but with different layer sizes. Since this simulation study classifies collections of sentences, Doc2Vec was used as the feature extraction method instead of Word2Vec. As shown in Figure 2, the implemented model is made up of a combination of separable layers following:

- SeparableConv1D: this is a one-dimensional convolutional layer, mostly used for text, that can be used to detect features in a vector. This type of layer will detect patterns and connections within the records. The ReLu activation accompanying this layer has been beneficial for training deep neural networks (Glorot et al., 2011). This layer has a size setting and a filter size setting (represented as K5 and K3). The size setting shows the number of filters (in this case 256), and the filter size represents the sliding window in the convolution layer, 5 by 5, and 3 by 3, respectively.
- Dropout: this type of layer is used as partial prevention for overfitting by setting a part of the nodes to 0 during each training step. Without Dropout, a node can correct behavior for another node during training. This corrective behavior can lead to overfitting because these fused nodes do not generalize to unseen data. Dropout prevents this from happening and thus reduces overfitting (Srivastava et al., 2014). Figure 2 shows what percentage of the nodes are dropped in each Dropout layer.
- MaxPooling1D: this layer reduces the network dimension size and generalizes patterns found by having kernels in the following layers by looking at relatively more data while keeping the same size.
- Dense: two Dense (or fully connected) layers are set up at the end of the CNN-based architecture, finalizing the network. These layers connect all patterns, which does not happen in the local-only convolutional layers. The number shown in Figure 2 represents the number of neurons.

As the size of the training data increases with each labeled record, so does the optimal amount of training epochs for the neural network. As a result, there is no universally optimal number of training epochs. A heuristic stopping rule was implemented to compensate for a fluctuating training data size. This rule is based on the network loss delta to avoid having under or overfit networks.

For a neural network to work best, it needs to be optimized. The settings steering the behavior of this convolutional neural network were empirically optimized using the GridSearchCV function found in the Scikit-learn library (REF). This grid search function cross-validates every setting five times^1^ and records network accuracy as a performance metric for each run. The following settings were available for optimization: batch size, early stopping patience, early stopping delta, dropout rates, optimization method, kernel size, and filter size. The settings with the highest network accuracy were implemented in the final model.

To adjust for the possible sparsity of a dataset, a convolutional neural network usually adjusts its weights based on class imbalance. The implemented CNN in this study was modified not to calculate a class weight, as the ASReview software has an integrated balancer, making rebalancing the class weights redundant.

The implemented convolutional neural network is built from combinations of these dense neural network layers, separable convolutional layers, activation layers, pooling layers, and dropout layers. The resulting 17 hidden layers deep architecture shown in Figure 2 is published on GitHub and Zenodo (Teijema, 2021a) as a plugin for ASReview.

As this network can handle a wider input size (as a result of being more computationally efficient), a companion feature extractor was created based on the current doc2vec implementation. Doc2vec can be a powerful feature extractor but fails to capture out-of-vocabulary words (Naseem et al., 2021). The standard doc2vec implementation has a vocabulary size of 40. The new feature extractor will be a wider doc2vec implementation with different vocabulary size. The vocabulary size for the new wider doc2vec feature extractor was set to 120 after 5-fold cross-validation in WSS@100% performance using 80, 120, and 250 as potential vocabulary sizes. This resulted vocabulary size should not be taken as universal vocabulary size but rather as near optimum for this dataset. This wider doc2vec v0.1.2 is available as a plugin for the software ASReview (Teijema, 2021b).

### 3.2. Results

The performance of the CNN is evaluated in the subsequent two studies by using it as a stand-alone classifier and a switch-to model for switching performance. It will be compared using the 95 and 100% WSS metrics. In response to RQ2.1, our findings demonstrate that a convolutional neural network can be implemented effectively in text classification for active learning in systematic reviews.

## 4. Study 3—Performance and computation time

The third study compares the classifier performance in terms of work saved over sampling (Cohen et al., 2006) for different combinations of classifiers with feature extraction techniques and compares these combinations with the newly developed 17-layer CNN model. In this study, we aim to answer the following two questions:

**RQ3.1:** Which combination of feature extraction technique and classification method gives the best performance in terms of WSS for the Brouwer et al. dataset?

**RQ3.2:** How do the available models compare in terms of computational time and performance?

### 4.1. Method

All possible combinations of feature extraction techniques and classification methods are used in different simulations using the Brouwer dataset. Those simulations are then analyzed for performance and computational statistics. Computational time is presented for the feature extractor and the average iteration time. The order in which records are found in the simulations is registered, and a correlation between this order is calculated for each model.

### 4.2. Setup

This study combined all classifiers (naive Bayes, logistic regression, random forest, support vector machine, and a 2-layer neural network) with feature extraction techniques (TF-IDF, Doc2Vec, and SBERT) available in ASReview v0.18, plus the CNN model developed in Study 2. Only viable combinations were tested as it is impossible to test naive Bayes in combination with doc2vec and SBERT because the multinomial naive Bayes classifier cannot handle matrices containing negative values, which these feature extraction strategies generate in their representations. Moreover, the combination of a neural network and TF-IDF is not feasible because the feature matrices produced by TF-IDF are too wide to realistically employ in the implemented neural network due to limitations in working memory. The remaining combinations were used for simulations.

The results from study 1 show that the performance for simulations with different prior records is very similar, with a low standard deviation in performance. Based on these results, only 1 set of priors for the subsequent simulations was picked through a simulation seed. Furthermore, as study 1 found the last-to-find records of no particular relevance, and since human screening misses 10% of records on average, classifiers are compared at a WSS of 95%, judging performance more similar to real-world application.

The simulations were terminated when all relevant publications were found to save computational time. Running the simulations further would not influence the results, and termination reduces the computational time required to finish the simulation. Each simulation was initiated with 20 records of prior knowledge; ten included records and ten excluded records. The selected prior knowledge was the same for each simulation.

Note that while saving computational time, terminating after all relevant records are found is not representative of any behavior in a real active learning-based systematic review, as it is unknown when all relevant records are found.

### 4.3. Results

**Figure 3:**
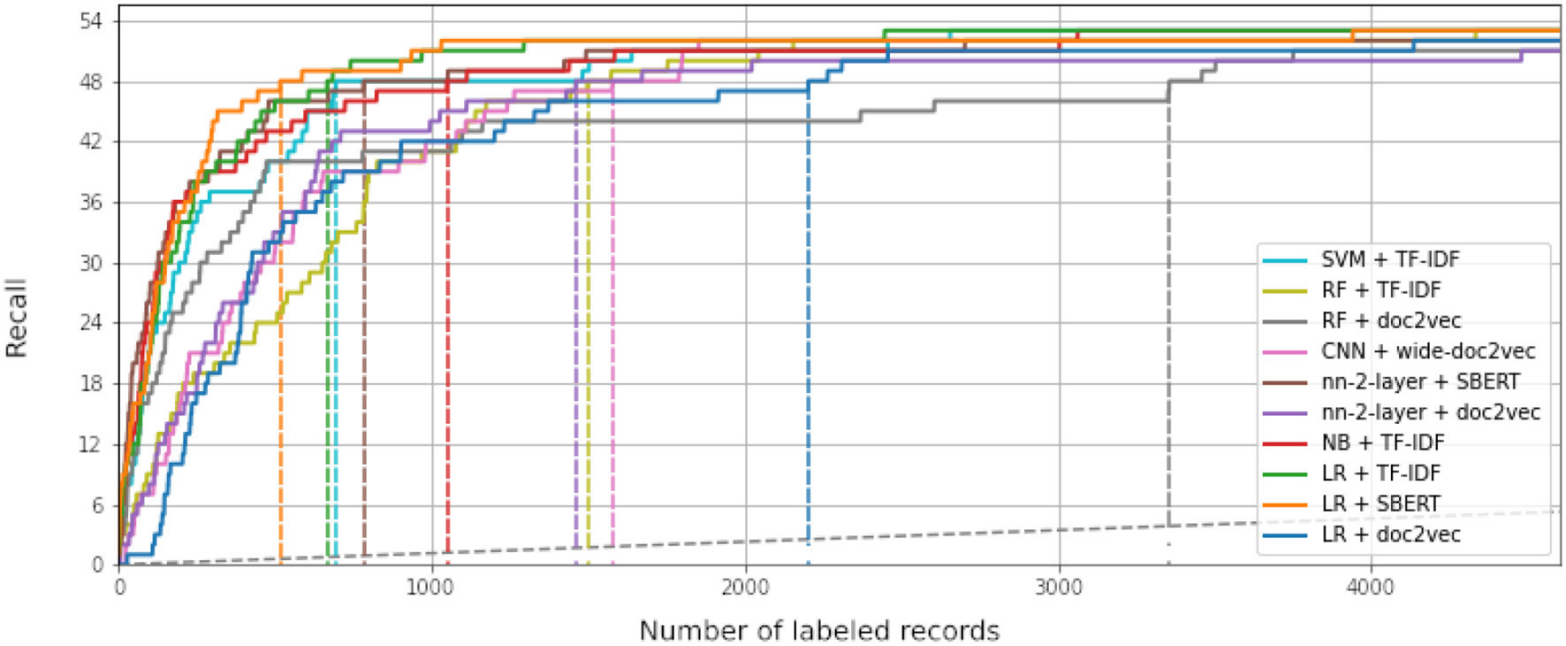
Recall curves for each of the simulation runs performed in this study. The x-axis shows the number of screened records. It is cut off after 4,000 records, less than 10% of the total amount of available records. The y-axis represents the found relevant records. The dataset contains 63 relevant records in total, and ten were given as prior knowledge, making the relevant record axis in this figure go up to 53 records. The dotted gray line represents random reading. The colored dotted lines represent the WSS@95% for each simulation. LR, logistic regression; SVM, support vector machine; NB, naïve Bayes; RF, random forest; nn-2-layer, 2 layers deep neural network; CNN, 17 layers convolutional neural network; TF-IDF, term frequency-inverse document frequency; SBERT, Sentence-BERT. Each simulation was cut off after all relevant records were found (*n* = 4000).

**Figure 4:**
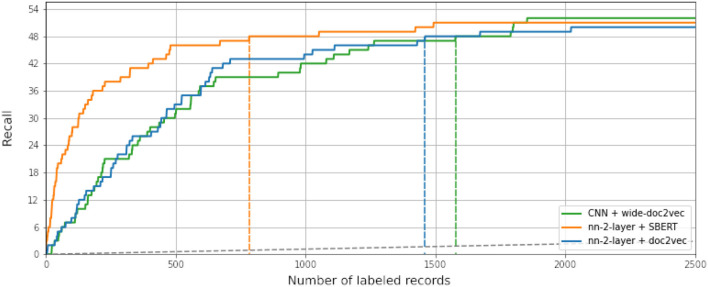
Neural network comparison at WSS@90%. The convolutional neural network with the wider doc2vec implementation and the two-layer neural network with both SBERT and doc2vec as feature extractors. When compared in finding the last record, only the convolutional neural network finds these records before the cutoff.

**Figure 5:**
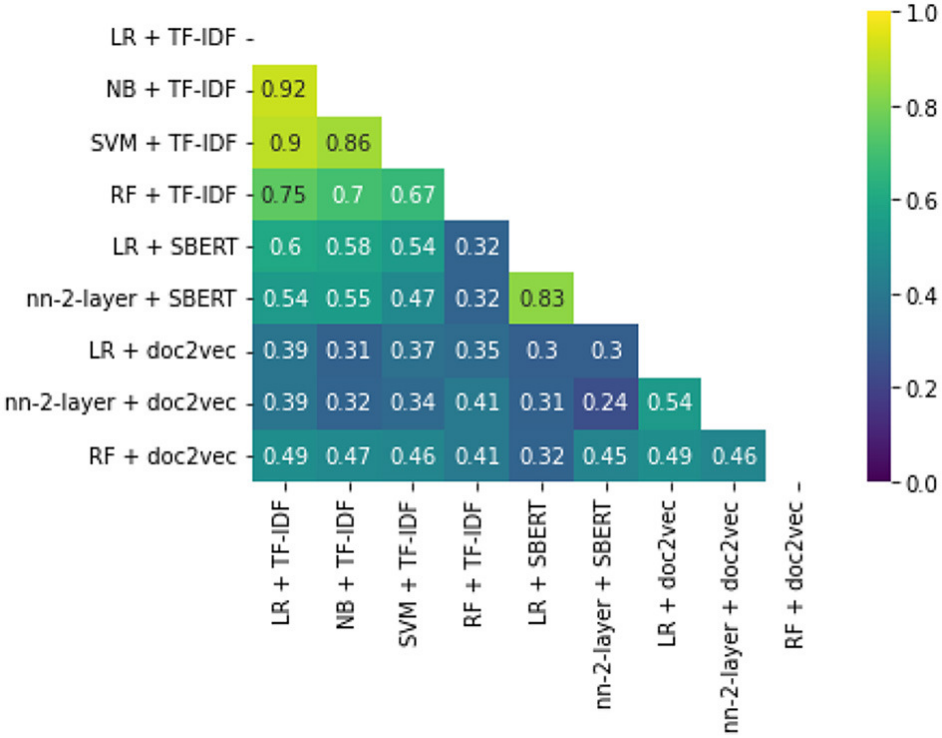
Rank order cohesion correlation matrix. This figure shows how similar or different the order can be between models.

While some combinations perform better than others, all simulations outperform random reading significantly. The simulation with the highest WSS@95% used Logistic Regression as a classifier, combined with SBERT as a feature extractor. This model combination found 95% of all records after screening 587 records, only 1.3% of all records. For comparison, on average with random reading, only one relevant record is expected to be found for every 750 screened papers. The recall of models can be seen in Figure 3, and the WSS@95 is provided in the first column in Table 1. To zoom in on the neural network models, we isolated the recall of these three models in Figure 4. As can be seen, the deeper network starts to outperform the lighter networks only at the very end of the simulation, finding the last records significantly faster than the other models. The best performance is nn-2-layer + SBERT, finding the 48th record significantly faster than the other models. Figure 5 shows the correlation matrix of cohesion between the order in which records were found (the rank order) for different classifiers and feature extractors. Note how the correlation is lowest between feature extractors but high for classifiers. Therefore, the order in which records are found is different for each model and is mainly caused by the different feature extractors.

**Table 1:** Performance metrics for each simulation run.

**Classifier + FE**	**WSS@95%**	**Feature extractor time**	**Median iteration time**
LR + SBERT	94,21%	6:27:23.23	0:00:00.19
LR + TF-IDF	94,14%	0:00:23.35	0:00:00.05
nn-2-layer + SBERT	93,01%	6:58:30.89	0:00:02.79
NB + TF-IDF	92,81%	0:00:13.62	0:00:00.03
SVM + TF-IDF	92,69%	0:00:15.57	0:00:08.95
CNN + wide-doc2vec	92,34%	0:32:25.44	0:00:59.17
RF + TF-IDF	91,82%	0:00:15.56	0:00:02.45
LR + doc2vec	90,93%	0:18:01.80	0:00:00.02
RF + doc2vec	88,14%	0:15:42.61	0:00:00.57
nn-2-layer + doc2vec	86,57%	0:18:03.91	0:00:01.75

LR, logistic regression; SVM, support vector machine; NB, naïve Bayes; RF, random forest; nn-2-layer, 2 layers deep neural network; CNN, 17 layers convolutional neural network; TF-IDF, term frequency-inverse document frequency; SBERT, Sentence-BERT.

Table 1 shows the computational time for each model. The feature extractor vs. the iteration time difference in computational time can be found. Especially, sBERT significantly increases computational time, followed by doc2vec. Most shallow classifiers are done by training a new model in a split second, and, as expected, the CNN takes much longer.

Our results address RQ3.1 and RQ3.2 as follows: The best-performing combination of feature extraction technique and classification method in terms of WSS for the Brouwer et al. dataset was Logistic Regression with SBERT as a feature extractor, achieving a WSS@95% of 94.21%. In terms of computational time and performance, we found that SBERT significantly increased computational time, followed by Doc2Vec. Most shallow classifiers trained new models quickly, while the CNN took much longer. Notably, the deeper neural network started to outperform the lighter networks only at the very end of the simulation, finding the last records significantly faster than the other models, with the best performance being the 2-layer neural network with SBERT. The order in which records were found varied for each model, mainly caused by the different feature extractors.

## 5. Simulation study 4—Model switching

The fourth study investigates the performance of the models when switching from one model to another, aiming to create a form of artificial paradigm shift. As different models struggle with different records, switching models might increase the performance of the pipeline. A re-representation of the information should have a significant transformative value for the machine learning algorithm. Here, we aim to answer the following research question.

**RQ4.1:** Can the performance of the active learning pipeline improve by switching models during the live review process?

### 5.1. Method

This study uses simulations of different machine learning models to investigate whether switching models during the active learning process can improve performance. An ASReview extension was developed to switch between models after a manually set number of records have been screened. The study compares the number of relevant records found after a certain percentage of screened records in the switched simulations to the values of the results of simulation study 3.

### 5.2. Setup

In the fourth study, model simulations from the third study were terminated after a stopping heuristic was reached (e.g., 50 irrelevant records are labeled consecutively) and continued with a different model to investigate if this increases performance. For the simulations, naive Bayes and TF-IDF were selected because it is the default in the software, and Logistic regression with SBERT was chosen as it was the best performing model from study 3. In aid of this switching process, an ASReview extension was developed to switch between models after a manually set number of records have been screened (Teijema, 2021c).

To quantify the performance of models after switching, the number of relevant records found after 1% (464 records), 1.5% (696), 2% (928), and 2.8% (1,391) of screened records in the switched simulations are compared to the values of the results of simulation study 3. The metric used for this is Relevant Records Found. The RRF@X% value represents the number of records found after X% of records are screened. The RRF values for switched simulations take this into account and thus represent X% of screened records, including those screened before switching.

### 5.3. Results

**Figure 6:**
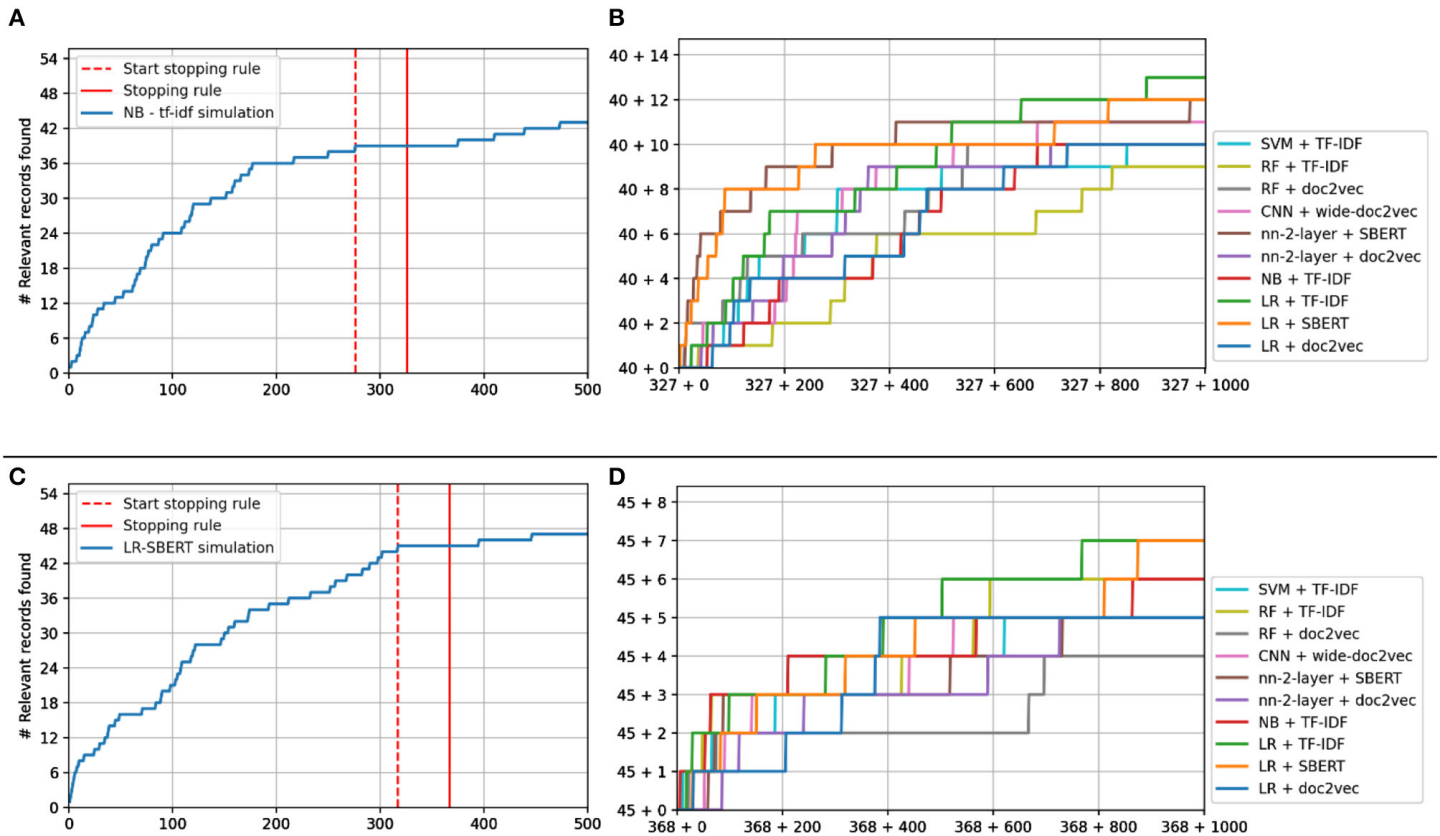
Study 4 simulation results. **(A, C)** Show the recall curve for the simulation starting as Naïve Bayes **(A)** and logistic regression **(C)** before and after switching. **(B, D)** Show the recall plots of the other models after switching.

Figure 6 shows the performance of switching models from the original model. NB + TF-IDF and LR + SBERT serve as benchmark values since, in those simulations, the model was not switched from the starting model.

The stopping rule was triggered at 326 records for the Naïve Bayes simulation, having found 40 of 53 records at that point, see Figure 6A. For Logistic Regression, it was triggered at 367 records, having found 45 records, see Figure 6C. As can be seen in Figure 6B, switching from Naïve Bayes + TF-IDF to a different model almost always results in a performance increase, especially when a different feature extractor is selected. For LR + SBERT, the results are less different since continuing with LR+SBERT already has the best optimal performance.

**Figure 7:**
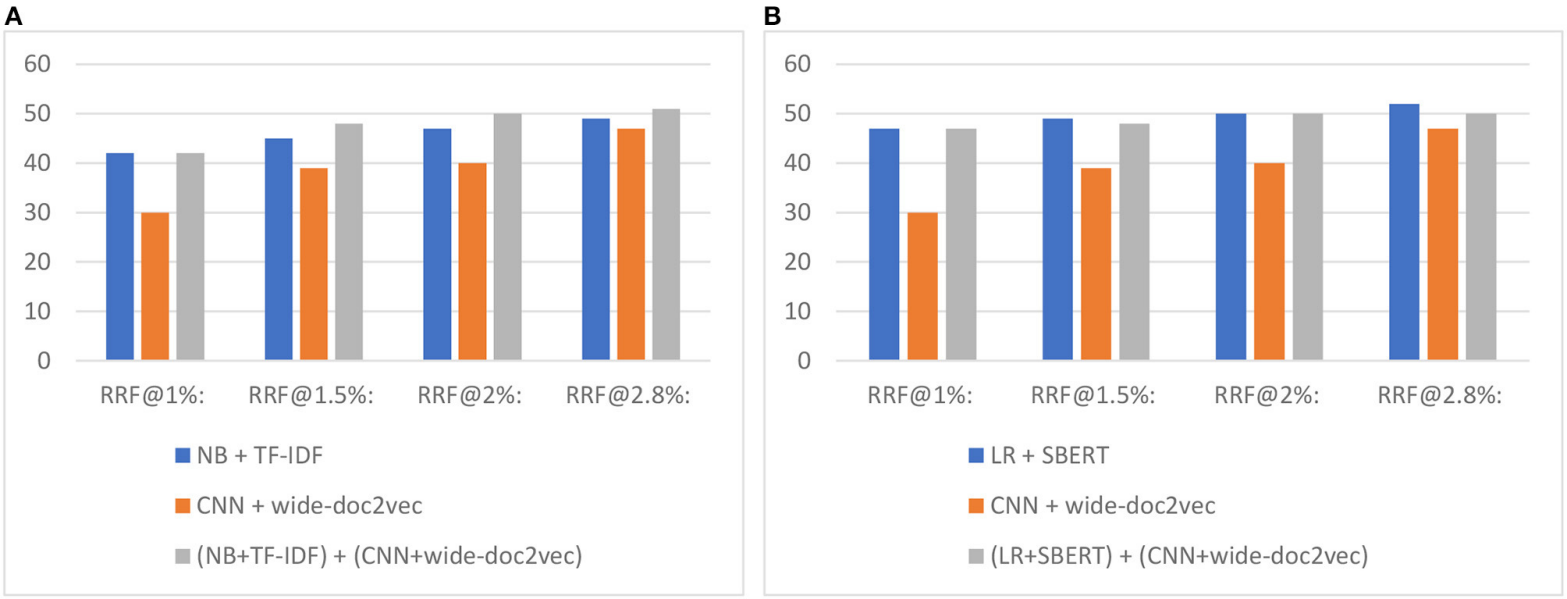
Study 4 switching results for the 17-layer CNN model combined with **(A)** Naïve Bayes and **(B)** logistic regression. The value on the y-axis is the total number of records found. The RRF@X% represents the number of found relevant records after screening X%.

Figure 7 shows *the total number of relevant records found after screening X% of records* when switching to the CNN model. As can be seen, and as expected, first running a shallow model and then switching to the CNN model outperforms only screening with the CNN model.

**Figure 8:**
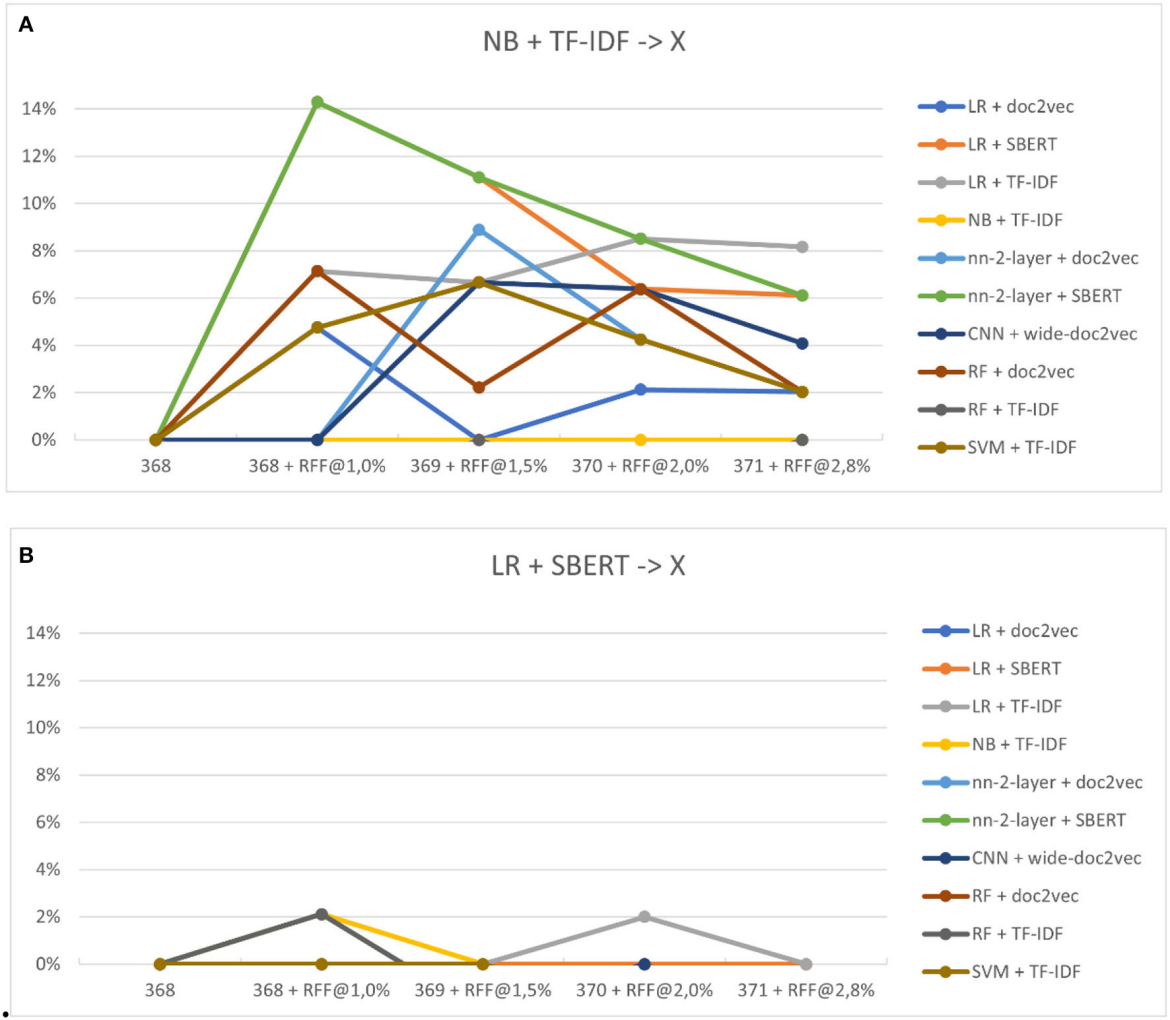
**(A, B)** The relative performance of classifiers after switching. **(A)** Shows the performance of switching from Naïve Bayes to other models (x). **(B)** Shows logistic regression to other models (x).

Figures 8A, B show the performance increase due to switching to a different classifier. Switching classifiers seems to outperform the default Naïve Bayes classifier for nearly every model, even by models that performed worse than Naïve Bayes in study three. No average improvement is found relative to the optimal classifier, Logistic Regression. However, models outperform even logistic regression in certain steps, whereas LR was superior in every situation previously. Note that in real review situations, the optimal model is unknown, and the selection for Naïve Bayes is more likely the selected classification model.

For RQ4.1, our results from the fourth study demonstrated that the performance of the active learning pipeline can indeed improve by switching models during the live review process. Specifically, we found that switching from Naïve Bayes + TF-IDF to a different model almost always resulted in a performance increase, especially when a different feature extractor was selected. Although there was no average improvement found relative to the optimal classifier, Logistic Regression, some models outperformed even logistic regression in certain steps, whereas LR was superior in every situation previously. This suggests that model-switching can enhance the active learning pipeline's performance in the live review process.

## 6. Discussion

The main goal of this study was to analyze performance in active learning-aided systematic reviews for a newly developed deep learning-based model compared to traditional machine learning approaches and investigate if switching between these models increases performance. The paper was divided into four different studies.

The goal of the first study was to analyze the performance and stability of the active learning-aided systematic review. The average time to discovery (ATD) was calculated for each record, and then the original meta-analyses were re-analyzed, excluding the 5 and 10% of the records with the highest ATD. Scenarios excluding the last-to-find records, the meta-analysis would have concluded with the same results for almost all topics. Overall, the results remained alike for the analyses on time to relapse (Hazard Ratio) and primary analyses (pooled effect sizes for the five leading theories).

The screening is terminated when using active learning before all records are screened. While simulations show that terminating has a very low chance of excluding relevant records, the only way to an absolute certainty is to screen all records (assuming 100% manual screening accuracy). Results from the first study show that last-to-find records (and thus those most likely to be missed) are not last to find due to their importance and that the impact of terminating is minimal. Even the most last-to-find records are found after screening < 10% of total records in the simulations. Finally, random screening by humans misses on average about 10% of records as a result of, among others, screening fatigue. Considering that screening fatigue is reduced when using active learning, the number of missed records by human error is reduced. Combining these results supports the use of active learning-based systematic reviews over random screening-based systematic reviews, saving screening time without sacrificing quality.

The second study implemented a convolutional neural network as a classifier for active learning-based systematic reviews. The implementation was made open source and is available online. In support of this model, the study implements a specialized doc2vec feature extractor. The performance of this model was measured in the third study.

The third study provides a performance overview for available classifiers and feature extractors. While these results should only be interpreted in the context of the selected dataset, it shows that while performance is generally good, there is a notable performance gap. Choosing the best models for a dataset is critical, as even minor performance differences can save work hours. The results show significant differences in computing time for classifiers and feature extractors. The results can be found on the Github page (Teijema et al., 2022).

Regarding performance, the results show that the default model of NB + TF-IDF in ASReview v0.18 is only fourth on the fastest combination available. LR + SBERT was the best performing combination for this dataset, finding 95% of all relevant records in only 587 screened records. The performance of LR + TF-IDF was a close second. As the computational time for TF-IDF is significantly lower, the results of this paper show that LR + TF-IDF was the best choice of model for this dataset. Whether or not this result is unique to this dataset or universal should be the topic of future work. Only after empirical testing can a suggestion for a new default model be given.

The study compared the performance of different neural network-based classifiers. It showed that while the smaller networks are quicker in finding the bulk of the relevant records, the deeper convolutional neural network is the first and only to find all relevant records before termination.

Suppose the last-to-find record is indeed last due to being farther removed from the other relevant records in terms of content. In that case, the deeper convolutional neural network is expected to have the best performance in finding it, as it was designed to identify more complex patterns. Whether or not the last-to-find record is different in content and distinct from other records is the topic of further work.

Interesting is that neural networks usually only perform well when the dataset contains millions of samples. In our case, only a small amount of samples what available, and still, the network performed well.

Finally, the order in which classifiers find relevant records was compared on order correlation. It was found that the lowest correlations are found between feature extractors rather than classifiers. The feature extractor indicates which information is gathered from each record, creating a hidden network of patterns. The classifiers' job is to sort out which patterns are relevant as quickly as possible. The shape of the resulting network of classified hidden patterns is thus more dependent on the feature extractor than the classifier, even though the WSS performance is relatively more dependent on the latter. This phenomenon follows from the third study. It shows that WSS@95% performance is not dominated by either classifier or feature extractor, where correlation is highly dependent on the feature extractor.

Study four shows the performance of switching from one model to another after a set heuristic switching rule was reached. Performance was measured for switching from the default model and for the best-performing model found in study 3. The models were switched to every available model, including the newly developed convolutional neural network in study 2.

The expectation for the fourth study was that lighter models perform best in the early stages of the simulation, while other simulations have increased performance in later stages. This was indeed observed in the results, as models that previously would not outperform the lighter models now suddenly performed equal or better than from the start.

Naïve Bayes with TF-IDF performed average in the simulations from the third study, being the fourth-fastest model on average. However, when measured from the switching point onwards, almost every model outperformed Naïve Bayes + TF-IDF. From that point, the original NB model was on par with the worst-performing models found in the simulations of study three.

The optimal model from study three was logistic regression. Even this model was outperformed by other switched-to models at certain steps. In the simulations found in study three, LR was superior in every step. Note that in systematic reviews, the optimal model is unknown. It is unlikely that the optimal model is selected from the start, and the default model is more likely to be chosen. This paper found that, on average, switching models is the preferable choice when the optimal is unknown.

### 6.1. Limitations

Based on the study presented in the paper, we have identified the following major challenges in the domain of active learning-aided systematic reviews:

- Model selection and performance: Choosing the best models for a dataset is critical, as even minor performance differences can save work hours. While the performance of different models was assessed in this study, further investigation is needed to determine if the results are unique to this dataset or universally applicable.
- Re-training frequency: Determining the optimal frequency for re-training a model is a significant challenge. The difference in performance between re-training with every newly found record, every n amount of records, or even training only once remains unclear.
- Training time trade-offs: Balancing training time and model performance is crucial. In practice, if a model takes longer to train, it can lead to skipping training iterations. This might lead to a fast training model potentially outperforming a slower but better model in certain situations.
- Optimal model switching point: Identifying the optimal point in the active learning process for model switching is a challenge. This point might be when the order of records does not change any further, but further investigation is required to verify this.
- Contentual differences in record findability: Understanding how contentual differences in records impact their findability and rank order can help improve active learning-aided systematic review performance. This aspect requires further research.
- Generalizability of results: The simulation results of this study are specific to the Brouwer dataset and cannot be directly applied to other datasets. For generalizable results, a benchmark platform comprising several different datasets with divergent topics and characteristics is suggested to empirically compare the performance of different models.

### 6.2. Future work

For future datasets like the depression disorder dataset used in this study, researchers can use active learning to their advantage by leveraging its ability to expand their original search. Active learning reduces the screening effort, allowing researchers to screen more records and potentially identify more relevant records. Furthermore, as shown in this study, switching classification models can be beneficial in improving the performance of the active learning-based systematic review. Hence, future researchers should consider incorporating model switching strategies into their active learning-based systematic review workflow to achieve better performance. Finally, further research can investigate the application of active learning in other mental health research areas, such as anxiety disorders or substance abuse, to explore its potential benefits in these domains.

## 7. Conclusion

The main conclusion from this study is that models have a preferred simulation stage in which the model performs best. Some work better in the early stages of the review, while others shine in the later stages of the simulation. This behavior is most apparent in heavier models like the two layer deep neural network and the convolutional neural network. These models go from being among the worst to top-performing models when applied correctly in the later stages of the simulation.

Considering the results of this study leads to a strong suggestion for the switching-model use case. On average, switching models increases performance over the default classification model. In future applications of active learning-based systematic reviews, ensemble models or hybrid models could replicate the results from this research. Until then, the current advice is to start the review with a light model such as the Naïve Bayes classifier or logistic regression and set a heuristic rule (such as labeling 50 irrelevant models in a row) as a point to switch to a heavier classification model.

## Data availability statement

The datasets presented in this study can be found in online repositories. The names of the repository/repositories and accession number(s) can be found below: https://doi.org/10.5281/zenodo.6799805.

## Author contributions

Main author: JT. Feedback and rewriting passages: JT, LH, JdBr, RvdS, and AB. Dataset development: MB, JdBr, RvdS, GF, JdBo, and CB. Method development: JT, AB, and SvdB. Dataset screening: SvdB. Code development: JT and PV. All authors contributed to the article and approved the submitted version.

## Footnotes

^1^Five-fold cross-validation is the default setting in scikit-learn following version 0.22, which is widely adopted because it strikes a good balance between bias and variance. This helps to mitigate overfitting and ensures that the model generalizes well to new data.
